# Comparison of Rapid Diagnostic Tests for the Detection of *Plasmodium vivax* Malaria in South Korea

**DOI:** 10.1371/journal.pone.0064353

**Published:** 2013-05-07

**Authors:** Jung-Yeon Kim, So-Young Ji, Youn-Kyoung Goo, Byoung-Kuk Na, Hyo-Joo Pyo, Han-Na Lee, Juyoung Lee, Nam Hee Kim, Lorenz von Seidlein, Qin Cheng, Shin-Hyung Cho, Won-Ja Lee

**Affiliations:** 1 Division of Malaria and Parasitic Diseases, National Institute of Health, Korea CDC, Osong, Republic of Korea; 2 Department of Parasitology and Tropical Medicine, Kyungpook National University School of Medicine, Daegu, Republic of Korea; 3 Department of Parasitology and Institute of Health Sciences, School of Medicine, Gyeongsang National University, Jinju, Republic of Korea; 4 Division of Structural and Functional Genomics, Center for Genome Science, National Institute of Health, Korea CDC, Osong, Republic of Korea; 5 Global Health Division, Menzies School of Health Research, Casuarina, Northern Territory, Australia; 6 Drug Resistance and Diagnostics, Australian Army Malaria Institute, Brisbane, Australia; National University of Singapore, Singapore

## Abstract

South Korea is one of many countries with endemic *Plasmodium vivax* malaria. Here we report the evaluation of four rapid diagnostic tests (RDTs) for diagnosis of this disease. A total of 253 subjects were enrolled in the study. The sensitivities, specificities and agreement frequencies were estimated by comparing the four RDTs against the standard of nested-PCR and microscopic examination. The CareStart^TM^ and SD Bioline had higher test sensitivities (99.4 and 98.8%, respectively) compared with the NanoSign and Asan Easy tests (93.0 and 94.7%, respectively). The CareStart^TM^ and SD Bioline tests could detect *P. vivax* in samples with parasite densities <150/μl, which was a slightly better performance than the other two RDTs. The quantitative accuracy of the four RDTs was also estimated by comparing results with *P. vivax* counts from blood samples. Lower test price would result in increased use of these RDTs in the field. The results of this study contribute valuable information that will aid in the selection of a diagnostic method for the detection of malaria.

## Introduction

Worldwide, *Plasmodium vivax* accounts for an estimated 80 million cases of malaria each year [1]. *P. vivax* is the only indigenous *Plasmodium* species in South Korea. Vivax malaria has been endemic in Korea and seasonal transmission has occurred since the disease re-emerged in 1993. From 1994 to 2010, 29,692 cases of endemic vivax malaria were reported. South Korea has embarked on a malaria elimination program based on early and accurate diagnosis followed by radical and rapid treatment. Standardization and use of sensitive diagnostic tools are critically important for a successful program outcome.

During the last 100 years, malaria has been diagnosed by microscopic examination of Giemsa-stained thick and thin blood films [2], [3] and today this approach is the gold standard for malaria diagnosis that is recommended by the World Health Organization (WHO). However, microscopy performed by poorly trained personnel that live in the rural areas of endemic malaria has a low sensitivity [4], [5]. This limitation has prompted research and development of reliable, easy to perform, rapid tests such as Rapid Diagnostic Test (RDT) to detect the presence of malaria parasites at levels of accuracy comparable to skilled microscopists. RDTs use immunochromatographic techniques to identify *Plasmodium* antigens. RDT performance is affected by the target antigen [6], [7]. RDTs are being used to diagnose vivax malaria in field studies [8], [9], as an alternative to microscopy at health facilities where quality microscopy is not available. Indeed, diagnosticians depend on RDTs for malaria diagnosis in endemic regions and in soldiers of Korean armed forces.

RDT performance varies with the environment of employment, geographical location, disease prevalence, and parasite species. RDT sensitivity declines with low parasite densities (<300–500/μl) [9], [10]. To address this problem, WHO collaborated with the Foundation for Innovative New Diagnostics (FIND) to systematically compare the laboratory performance of RDTs. Four rounds of testing have been published (http://www.finddiagnostics.org/resource-centre/reports_brochures/malaria-diagnostic-test-report.html). Interestingly, some field test results have differed from the laboratory results reported by WHO/FIND [11], which highlights the importance of testing RDTs in different environments. For example, WHO/FIND reported good performance for the Opti-MAL rapid malaria test, but the test performed significantly worse in a Thailand study [12]. From 2009 to 2011, we evaluated the performance of four RDTs at the National Laboratory, Korea. Three of the RDTs (SD Bioline, NanoSign and Asan Easy) are produced in South Korea and one test (CareStart^TM^) is produced in the United States. The SD Bioline and CareStart^TM^ tests performed well in the WHO/FIND comparison [13], [14]. A total of 171 fresh blood samples from patients diagnosed with vivax malaria at local health centers (2009–2011), and 82 blood samples from asymptomatic and aparasitemic healthy volunteers were used in this evaluation. RDT performance was compared to microscopic examination and nested PCR. The accuracy of each RDT for the quantification of parasite density was also investigated.

## Materials and Methods

### Ethics statements

The study was approved by the ethics committee of the Korean National Institute of Health. An approval form was used to obtain written informed consent from each participant. Each participant also gave permission to provide a 5 ml blood sample.

### Sample collection

**Figure 1:**
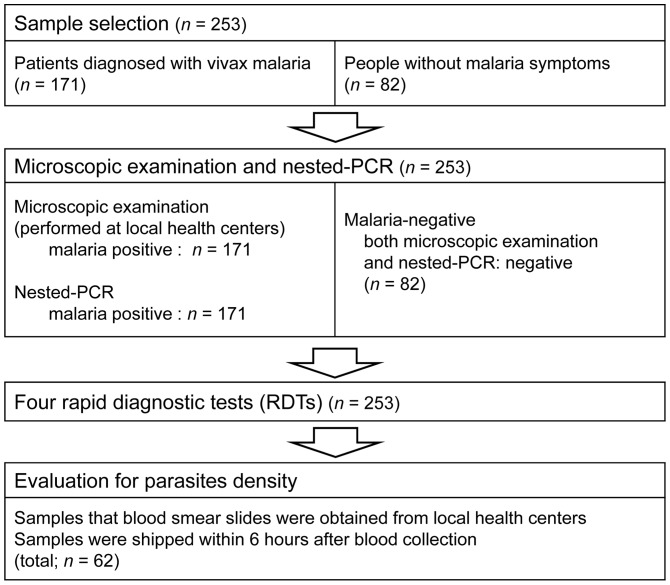
Sample selection profile for the evaluation of four rapid diagnostic tests. Microscopic examination, nested-PCR, and rapid diagnostic tests were performed.

The study was conducted at the Division of Malaria and Parasitic Diseases, the Korea Centers for Disease Control and Prevention (KCDC), from June 2009–October 2011. Venous blood samples with EDTA were received from 171 patients diagnosed with vivax malaria at local health centers (Kang-wha, Paju, Gimpo, Yeon-Cheon; Fig. 1). Microscopic examination of Giemsa-stained thick and thin blood films was used to confirm the diagnosis. Samples were also received from 82 asymptomatic and aparasitemic healthy volunteers, which had been confirmed as *P. vivax* negative by microscopic examination and nested-PCR. Mean age of the patients was 47 years, and 75% of them were male. Blood specimens were kept cool and arrived at the KCDC within 24 hours after collection.

### Characterization of rapid diagnostic tests

All blood samples were assayed with each of four commercial RDTs. CareStart^TM^ Malaria HRP/pLDH (Access Bio, USA) detected parasite antigen *Pf*HRP-2 specific for *P. falciparum* in one capture site and pan-pLDH, which detected all four *Plasmodium* species, in a separate capture line. Individually packed test cassettes and fixed quantified sample buffer in a disposable tube were provided in each package of tests.

SD Bioline Ag P.f/pan Rapid (SD, Korea), also in individually packed test cassettes, detected parasite antigen *Pf*HRP-2 using a method similar to CareStart^TM^. However, sample buffer was supplied in a stock bottle and was not included in each package of test cassettes.

NanoSign Malaria P.f/P.v (Bioland, Korea), a device-type test with one capture line specific for *P. falciparum* (aldolase detection) and a second line that detected all *Plasmodium* species (pan-pLDH antigen).

Asan Easy Test^®^ malaria P.f/P.v strip (Asan Pharmaceutical, Korea), 48 packed strip kits used *Pf*LDH antigen (for *P. falciparum*) and *Pv*aldolase (for *P. vivax*).

The laboratory performance of the CareStart^TM^ Malaria HRP/pLDH, the SD Bioline Ag P.f/pan Rapid, and the NanoSign Malaria P.f/P.v tests has been evaluated by the WHO/FIND product testing program and results are available on the website (see above) [14].

Test results were recorded as negative if only a control band appeared. The presence of a control and test line indicated a parasite positive result. The test was considered invalid when a control band did not appear. RDT results were recorded by different technicians, but each sample was blinded against the results from the other diagnostic techniques and RDTs.

### Microscopic examinations

Microscopic examination was performed on 253 blood samples collected from vivax malaria patients (n = 171) and from healthy volunteers (n = 82). The 171 blood samples from patients with malaria symptoms were first examined microscopically at local health centers. In addition, blood smear slides for 62 of these samples were also obtained from local health centers or prepared at the KCDC for further quantitative study. Eighty-two samples from healthy volunteers were confirmed as malaria negative at the KCDC. Thick blood smears were prepared, dried, stained with 10% Giemsa, and examined microscopically. A slide was considered negative if no asexual stage of *Plasmodium* spp. were found during examination of 100 fields. Parasite densities were assessed by counting against 200 leucocytes, converting to parasites per microliter by assuming a standard leucocyte count of 8000/μl. Two technicians counted the parasites under study-blinded conditions.

### Nested polymerase chain reaction (Nested PCR)

Nested PCR amplification based on the 18S rRNA gene was performed for all blood samples as described by Snounou et al. [15] with slight modification. Using a DNA Mini kit (Qiagen, USA), genomic DNA was extracted from 200 μl of whole blood and was suspended in 200 μl of distilled water. Two microliters of DNA sample was added to a 18 μl master mixture containing 1 μl premix, 1 μl each primer (rPLU5 and rPLU6), and 15 μl distilled water. The PCR cycling conditions were: initial denaturation at 94°C for 5 min, 25 cycles of denaturation at 94°C for 30 sec, annealing at 60°C for 1 min, and extension at 72°C for 1 min. Two microliters of the first PCR product was used as a template for a nested PCR (internal primers rVIV1 and rVIV2). The nested-PCR cycling conditions were: initial denaturation at 92°C for 5 min, 25 cycles of denaturation at 92°C for 20 sec, annealing at 52°C for 20 sec, and extension at 78°C for 20 sec. The PCR products were resolved by electrophoresis on a 2% agarose gel, stained with ethidium bromide, and visualized with ultraviolet illumination.

### Data analysis

Estimates of sensitivity, specificity, and kappa were calculated with DAG_Stat and VassarStats (http://vassarstats.net/) [16]. Nested-PCR and microscopic examination results were used as the reference standard to estimate sensitivity and specificity, together with their 95% confidence intervals (CI) for the CareStart^TM^, SD malaria, NanoSign, and Asan Easy tests. The following kappa values were used to categorize the strength of agreement between the each RDT and nested-PCR and microscopic examination: fair (0.40 to 0.61), moderate (0.61 to 0.80), and substantial (>0.8). Whenever possible, Standards for the Reporting of Diagnostic Accuracy (STARD) guidelines were considered during preparation of this report [17].

### Quantitative performance of four RDTs

Blood samples from 62 patients whose parasite densities were determined by quantification of thick smears were used to assess the quantitative performance of the four RDTs. The positive (sample) line was divided into three groups depending on the ratio of the test line intensity compared to control line intensity (*: positive line < control line; **: positive line  =  control line; ***: positive line > control line). Three technicians read line intensity for all RDTs under conditions in which each sample was blinded against the results from the other diagnostic techniques. These results were compared with parasite counts obtained by thick smear. To facilitate ease of comparison, samples were divided into five groups based on the parasite densities: (1–150; 151–500; 501–1000; 1001–5000; >5000/μl).

## Results

### Diagnostic performance of four RDTs, nested PCR, and microscopic examination

Two hundred and fifty-three blood samples obtained from the malaria patients diagnosed at local health centers and healthy subjects were examined with the nested PCR and microscopy. One hundred and seventy-one of these samples tested positive for vivax malaria. The 82 healthy individuals tested negative for *P. vivax* (nested PCR; Table 1). The 171 vivax malaria positive samples were tested with each of the four RDTs; 170, 169, 159, and 162 of them were vivax malaria positive with the CareStart^TM^, SD Bioline, NanoSign, and Asan Easy tests, respectively. Results from the four RDTs for the 82 samples from healthy people were negative (Table 1).

**Table 1:** Performance of four rapid diagnostic tests when compared with microscopic examination for *Plasmodium vivax* malaria diagnosis.

Microscopic examination (n)	Nested-PCR		CareStart^TM^		SD Bioline		NanoSign		Asan Easy	
	Positive (%)	Negative (%)	Positive (%)	Negative (%)	Positive (%)	Negative (%)	Positive (%)	Negative (%)	Positive (%)	Negative (%)
Positive (171)	171(100)	0(0)	170(99.4)	1(0.6)	169(98.8)	2(1.2)	159(93.0)	12(7.0)	162(94.7)	9(5.3)
Negative (82)	0(0)	82(100)	0(0)	82(100)	0(0)	82(100)	0(0)	82(100)	0(0)	82(100)

### Sensitivity, specificity, and kappa value of four RDTs

RDT sensitivity and specificity were calculated using microscopic examination as the reference test. Sensitivity of the CareStart^TM^ (99.4%, CI: 96.3–100) and SD Bioline (98.8%, CI: 95.4–99.8) tests were greater than the sensitivities of the NanoSign (93.0%, CI: 87.8–96.2) and Asan Easy (94.7%, CI: 89.9–97.4) tests. Specificity was 100% (CI: 94.4–100) for all of the RDTs. Test agreement was calculated by comparing RDT results with microscopic examination. All RDTs showed high agreements (concordance: 95.3–99.4%, kappa value: 0.896–0.991) with microscopic examination (Table 2).

**Table 2:** Sensitivity, specificity and kappa value for rapid diagnostic tests relative to microscopy or nested-PCR for the detection of *Plasmodium vivax*.

RDT vs. PCR or Microscopy	Sensitivity (95%CI)	Specificity (95%CI)	Concordance (%)	Kappa value *
CareStart^TM^	99.4 (96.3–100)	100 (94.4–100)	99.4	0.991
SD Bioline	98.8 (95.4–99.8)	100 (94.4–100)	99.2	0.982
NanoSign	93.0 (87.8–96.2)	100 (94.4–100)	95.3	0.896
Asan Easy	94.7 (89.9–97.4)	100 (94.4–100)	96.4	0.921

All cases (n  =  256).

CI: confidence interval.

* The strength of agreement between each RDT and nested-PCR or microscopic examination: fair (0.41–0.60), moderate (0.61–0.80), and substantial (≥ 0.81).

### Quantitative performance of CareStart^TM^

**Figure 2:**
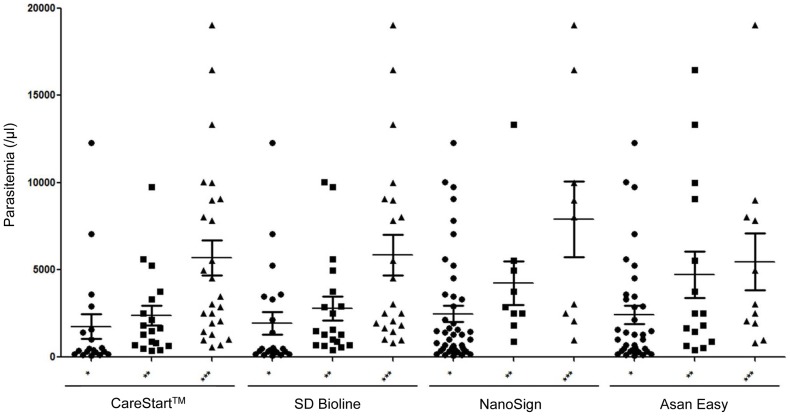
Correlation between intensity of the positive band of four rapid diagnostic tests (CareStart^TM^, SD Bioline, NanoSign, and Asan Easy) and parasitemia (determined by count of thick blood smears). ***; If the strength of positive line is greater than that of control line (Control line < Positive line), **; If the strength of the positive line is similar to the control line (Control line  =  Positive line), *; If the strength of the positive line is less than the control line (Control line > Positive line).

To determine whether the RDTs could detect low numbers of *P. vivax*, results for each sample were categorized according to parasite density. The 62 samples that were shipped with blood smears slides or prepared within 6 hours after blood collection were used for this evaluation. CareStart^TM^ and SD Bioline tests could detect *P. vivax* at low parasite densities (sensitivity: 100%), the NanoSign test had a low sensitivity (60%) at a parasite density of 1–150/μl. We wondered whether we could use RDTs to quantify the parasite load in the patient by comparing the ratio of test to control band intensity. Therefore, the ratio of test to control band intensity for the four RDTs was compared to corresponding parasitemia. There was a slight positive correlation between the ratio of the test to control band density for each RDT and the parasitemia counts (Fig. 2).

## Discussion

The WHO recommends microscopic examination as the gold standard for *P. vivax* malaria diagnosis. Physicians at local health centers still use this method, but have asked which of the RDTs is most accurate for diagnosis of this disease. In the Korean market, RDTs vary in price from US $4.40 to 6.60 per test, much more expensive when compared with the cost of these tests in other countries [14]. In this study, we compared the diagnostic performance of four RDTs relative to microscopic examination and nested-PCR to help physicians make an informed choice when selecting an RDT.

In the current study, CareStart^TM^, SD Bioline, NanoSign and Asan Easy, which are all currently used in South Korea, were selected for diagnosis of malaria caused *P. vivax*. In Round four of the WHO/FIND study, CareStart^TM^ and SD Bioline had high panel detection score for the diagnosis of vivax malaria at 94.1 and 97.1, respectively, when at least 200 parasites/μl were present. Few efficacy studies of NanoSign or Asan Easy test performance have been conducted, so the current study compared their performance with CareStart^TM^ and SD Bioline test. Vivax malaria is the only indigenous malaria in South Korea, and accounts for 99.9% of all cases without any history of travel abroad [18]. Therefore, although the pan-LDH line used in CareStart^TM^ and SD Bioline tests indicates the presence of at least one of three *Plasmodium* species (*P. vivax*, *P. malariae*, *P. ovale*), a definite pan-LDH line in samples from patients without a travel history to foreign countries could, at least initially, be considered to be vivax malaria positive.

As shown in Table 1, no case of plasmodium malaria was observed in 171 samples because the samples were collected near Demilitarized Zone (DMZ) regions where indigenous vivax malaria normally occurs. The sensitivities and specificities of the RDTs were higher than estimates from previous studies [9], [19]. In particular, sensitivities of the CareStart^TM^ and SD Bioline tests (99.4 and 98.8%, respectively) estimated by the present study were higher than previously reported by a hospital-based study in South Korea (95.5 and 92.7%, respectively) [19]. The difference may have resulted from the type of samples used in each study. In contrast with the present study, the previous study used frozen samples. Overall, the performance of the CareStart^TM^ and SD Bioline tests appears to be similar to microscopic examination and nested-PCR, which suggests that these RDTs could be used as alternative diagnostic tools for *P. vivax* detection. However, except for *P. falciparum*, these RDTs cannot differentiate *Plasmodium* species, which is disadvantageous for the diagnosis of imported malaria cases. Thus, we recommend that nested PCR should be performed after a positive CareStart^TM^ or SD Bioline test result to confirm and differentiate the *Plasmodium* species. These RDTs could be used for malaria screening and diagnosis at local health centers. When appropriate, samples could be sent to a laboratory with PCR capability for further diagnosis.

Because transmission rates and parasite densities in South Korea vivax malaria patients are usually low, a high RDT sensitivity is very important. As shown in Table 3, the four RDTs could detect vivax malaria in all samples at a concentration of >501/μl parasite density; however, each 3 and 1 vivax malaria samples were negative for the NanoSign and Asan Easy tests at a concentration of <500/μl parasite density. Although it is difficult to compare studies because of differences in parasite density, sensitivities of the CareStart^TM^ and SD Bioline tests at <150/μl parasite density (both 100%) were higher than previously reported [19]. Because there were a limited number of low parasitemia (<150/μl) samples in this study, additional studies would be required to confirm this result. Furthermore, additional quantitative study could be helpful for clinicians who would like to know the parasite load without preparing blood smears. As shown in Fig. 2, there was a slight correlation between parasite densities and the ratio of the test to control band density. However, there were many samples with high parasite densities but a smaller ratio (* in Fig. 2). Health center personnel could estimate the parasite load based on this ratio of the thickness of the control and test lines, but this estimate should be confirmed by microscopic examination for exact counting.

**Table 3:** Diagnostic performances of four rapid diagnostic tests compared with microscopic examination for various *Plasmodium vivax* parasite densities.

	No. of patients	No. of positive (Sensitivity%)			
		Care Start^TM^	SD Bioline	Nano Sign	Asan Easy
> 5000/μl	14	14 (100)	14 (100)	14 (100)	14 (100)
1001–5000/μl	23	23 (100)	23 (100)	23 (100)	23 (100)
501–1000/μl	9	9 (100)	9 (100)	9 (100)	9 (100)
151–500/μl	11	11 (100)	11 (100)	10 (90.9)	10 (90.9)
1–150/μl	5	5 (100)	5 (100)	3 (60.0)	5 (100)
Total	62	62 (100)	62 (100)	59 (95.2)	61 (98.4)

The malaria endemic regions of South Korea are restricted to near DMZ. Thus, 50% of patients are soldiers and discharged soldiers [18]. Some local areas, including some army camps in South Korea, have limited special staff trained to perform time-consuming microscopic diagnosis from examination of thick and thin smears. Use of RDTs could shorten the time to diagnosis.

Continuous product testing is necessary to guide personnel at local health centers and to improve RDT quality. The KCDC intended to summarize published data, including this study, to guide procurement decisions made by personnel at local health centers and hospitals in South Korea. Further, accurate and rapid malaria diagnosis may help to prevent secondary transmission in other countries. This study is expected to help improve the accuracy of malaria diagnosis in the field, especially under conditions of low parasitemia, and to assist with diagnostic method procurement decisions.
